# Habenular Neurogenesis in Zebrafish Is Regulated by a Hedgehog, Pax6 Proneural Gene Cascade

**DOI:** 10.1371/journal.pone.0158210

**Published:** 2016-07-07

**Authors:** Caroline Halluin, Romain Madelaine, François Naye, Bernard Peers, Myriam Roussigné, Patrick Blader

**Affiliations:** 1 Université de Toulouse III, UPS, Centre de Biologie du Développement (CBD), Centre de Biologie Intégrative (CBI), 118 route de Narbonne, F-31062 Toulouse, France; 2 CNRS, CBD UMR 5547, F-31062 Toulouse, France; 3 Stanford University, School of Medicine, 269–279 Campus Drive, Stanford, CA 94305, United States of America; 4 Unit of Molecular Biology and Genetic Engineering, University of Liège, GIGA-R, B34, Avenue de l'Hôpital 1, B-4000 Liège, Belgium; Institute of Cellular and Organismic Biology, TAIWAN

## Abstract

The habenulae are highly conserved nuclei in the dorsal diencephalon that connect the forebrain to the midbrain and hindbrain. These nuclei have been implicated in a broad variety of behaviours in humans, primates, rodents and zebrafish. Despite this, the molecular mechanisms that control the genesis and differentiation of neural progenitors in the habenulae remain relatively unknown. We have previously shown that, in zebrafish, the timing of habenular neurogenesis is left-right asymmetric and that in the absence of Nodal signalling this asymmetry is lost. Here, we show that habenular neurogenesis requires the homeobox transcription factor Pax6a and the redundant action of two proneural bHLH factors, Neurog1 and Neurod4. We present evidence that Hedgehog signalling is required for the expression of *pax6a*, which is in turn necessary for the expression of *neurog1* and *neurod4*. Finally, we demonstrate by pharmacological inhibition that Hedgehog signalling is required continuously during habenular neurogenesis and by cell transplantation experiments that pathway activation is required cell autonomously. Our data sheds light on the mechanism underlying habenular development that may provide insights into how Nodal signalling imposes asymmetry on the timing of habenular neurogenesis.

## Introduction

The Dorsal Diencephalic Conduction system (DDC) is a highly conserved neural circuit in the brain that consists of a pair of epithalamic nuclei, the habenulae, and two associated fibre tracts, the stria medullaris that carries axonal afferences from various area of the forebrain to the habenulae and the fasciculus retroflexus that conducts efferent axons from the habenulae to various midbrain/hindbrain target nuclei including the intrapeduncular nucleus or IPN [1,2]. The diversity in habenular afferent and efferent connections has led to the habenular nuclei being considered as major integrative and relay centres in the brain. Consistent with this idea, results from lesioning and electrophysiological studies indicate that the habenula complex is involved in controlling a multitude of cognitive functions and behaviours such as mating, aversive and olfactory guided behaviour, and spatial learning and attention [3,4]. More recently, the habenulae have caught neuroscientist’s attention for their role in the control of emotion and motivated behaviours. For instance, the lateral habenular subnucleus has been implicated in behaviour guided by error predictions in reward in primates and humans [5,6]. The medial subnucleus, on the other hand, has been shown to regulate anxiety and the expression of fear in zebrafish and mouse [7–9]. Finally, an abnormal increase in habenular activity has been associated with, and thought to drive symptoms of major depression in human [10] and in a rodent model of depression [11].

The habenulae display prominent left-right (LR) asymmetry in lower vertebrates, making them an attractive model to study the development of brain asymmetry [1,12–15]. In zebrafish, the habenulae consist of a dorsal (dHb) asymmetric and a ventral (vHb) symmetric domain that are homologous to the medial and lateral habenular sub-nuclei in mammals, respectively [16]. Based on the asymmetric expression of a restricted set of markers, the dHb has been further sub-divided into two main sub-nuclei whose sizes differ along the LR axis: the medial sub-nucleus (dHbm), which is bigger in the right habenula than the left and projects predominantly to the ventral part of the IPN, and the lateral sub-nucleus (dHbl), which is larger in the left habenula than the right and predominantly innervates the dorsal part of the IPN [17–21]. This subnuclear organisation implies that neuronal precursor of the dHb chose between at least two specification program (lateral or medial character) at different frequencies on the left and right sides. In zebrafish, this choice is predominantly controlled by a second epithalamic structure, the parapineal organ, a small nucleus that is specified at the midline and migrates to the left side in most wild type embryos [19,20]. While the orientation of habenular asymmetry always correlates with the side of parapineal migration, parapineal ablation prior to migration results in both habenulae adopting a predominantly “right” character [19,20]. Despite nearly 15 years of study, it is not yet clear how the parapineal imposes “left” character on the dorsal habenula, although recent studies indicate that this process involves a modulation of WNT signalling [22,23].

In addition to the role of the parapineal in the specification of habenular neuron identity, it has also been shown that there is an heterochrony in the birth of different habenular neuron subtypes; while habenular neurons are born first on the left and tend to populate the lateral sub-domain, neurons born later preferentially adopt medial characters [24]. We showed in a previous study that, in contrast to its requirement for later habenular asymmetries, the parapineal is not necessary for the early asymmetry in the timing of habenular neurogenesis [25]. We also showed that when a LR bias in Nodal signaling is abrogated, habenular neurons appear at the same time in both habenulae. Habenular neurons are nonetheless produced in the absence of Nodal activity leading us to propose that the Nodal pathway modulates a generic/symmetric programme of habenular neurogenesis, rendering it more effective/efficient on the left [25]. Relatively little is known about the mechanisms controlling this generic programme of habenular neurogenesis.

Here, we identify the proneural genes Neurog1 and Neurod4 as being redundantly required for habenular neurogenesis and show that their expression in the habenulae depends on the homeodomain transcription factor Pax6a. Furthermore, we show that habenular neural progenitors are absent in embryos mutant for the Hedgehog (Hh) receptor Smoothened, and that Pax6a, Neurog1 and Neurod4 act downstream of Hh signalling. Using a pharmacological inhibitor of Hh signalling, we show that pathway activity is required continuously for habenular neurogenesis. Finally, cell transplantation studies show that Hh pathway activation is required cell autonomously. Our results provide insights into the generic programme of habenular neurogenesis that will serve as a guide for understanding how Nodal signalling modulates asymmetric habenular development.

## Materials and Methods

### Fish lines and developmental conditions

Embryos were raised and staged according to standard protocols [26]. The transgenic lines *Tg(-8*.*4neurog1*:*GFP)*^*sb1*^ and *Tg(elavl3*:*EGFP)*^*knu3*^, and the *neurog1*^*hi1059*^, *smo*^*hi229*^ and *pax6b*^*sa86*^ mutations have previously been described [27–31]. Embryos homozygous for the various mutations were obtained by inter-crossing heterozygous carriers; adults heterozygous for the different mutant alleles were identified by PCR genotyping of tail-clip genomic DNA. Embryos were fixed overnight at 4°C in 4% paraformaldehyde/1xPBS, after which they were dehydrated through an ethanol series and stored at −20°C until use. Fixed embryos for *shha*^*tbx392*^ [32] and *smo*^*b641*^ [33] mutant lines were kindly provided by Simon Hughes (King College London, UK)

### Ethics statement

All animals were handled in a facility certified by the French Ministry of Agriculture (approval ID B-31-555-10) and in accordance with the guidelines from the European directive on the protection of animals used for scientific purposes (2010/63/UE), French Decret 2013–118. MR and PB have received an authorisation to experiment on vertebrates models (N° 311255556 and N° 311255553) from the ‘Direction Départementale de la Protection des Populations de la Haute-Garonne’. All anaesthesia and euthanasia procedures were performed in Tricaine Methanesulfonate (MS222) solutions as recommended for zebrafish (0,16mg/ml for anaesthesia, 0,30 mg/ml for euthanasia). All efforts were made to minimize the number of animals used and their suffering, according to the guiding principles from the Decret 2013–118.

### Cyclopamine treatment

Dechorionated embryos were treated at 16, 18, 20 and 24 hpf with 100μM cyclopamine (Toronto Research Clinical) by diluting a 10 mM ethanol-based stock solution in E3 medium. Control embryos were treated simultaneously with an equal volume of ethanol diluted 1/100 in E3 medium. Embryos were incubated at 28.5°C in cyclopamine continuously beginning at indicated time points until they were collected for processing.

### In situ hybridization and immunostaining

In situ hybridizations were performed as previously described [34]. Antisense DIG labelled probes for *brn3a* [17], *cxcr4b* [35], *pax6a* [36], *neurog1* [37] and *neurod4* [38] were generated using standard procedures. In situ hybridizations were revealed using BCIP and NBT (Roche) or Fast Red (Roche) as substrate. Immunohistochemical stainings were performed as previously described [39], using either anti-GFP (1/1000, Torrey Pines Biolabs) or anti-HuC/D (1/500, Molecular Probes); secondary antibodies used were Alexa 488 or Alexa 555-conjugated goat anti-rabbit IgG or goat anti-mouse IgG (1/1000, Molecular Probes). For nuclear staining, embryos were incubated in ToPro (1/1000, Molecular Probes) as previously described (25).

### Antisense morpholino injection and Transplantation

For morpholino knock-downs, embryos were injected at the one cell stage with either two previously described morpholinos targeting the 5’ end of *neurod4* [38] or a splice-blocking morpholino (GAGCACAGGTATTCTCCTCACCTGC) that targets the exon5/intron5 boundary of *pax6a*. Transplantation experiments were performed as previously described [39].

### Image acquisition

Bright field pictures were taken on a Nikon eclipse 80*i* microscope. Confocal acquisitions were acquired using a Leica SP5 or SP8 and confocal stacks were analysed using ImageJ software. Images were manipulated using Photoshop (Adobe) software.

## Results

### Habenular neurogenesis requires the redundant activity of *neurog1* and *neurod4*

**Fig 1:**
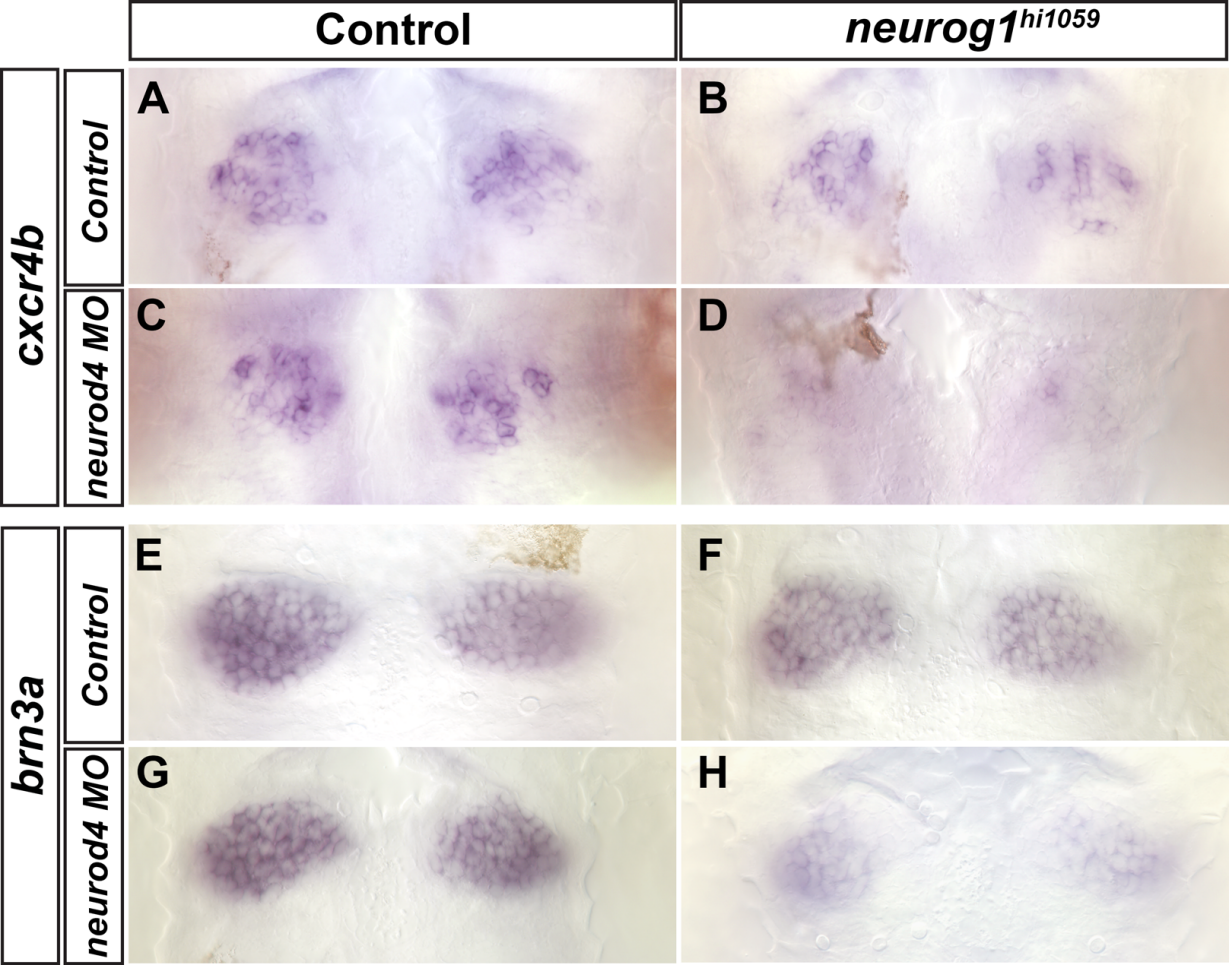
Combined loss of Neurog1 and Neurod4 function disrupts habenular neurogenesis. Whole-mount *in situ* hybridization against *cxcr4b* (A-D) or *brn3a* (F-H) showing the epithalamus of wild type (A,E), *neurog1*^*hi1059*^ mutant embryos (B,F), *neurod4* morpholino injected embryos (C,G) or *neurog1*^*hi1059*^;*neurod4* morphant embryos (D,H) at 36 (A-D) or 48 (E-H) hpf. The expression of *cxcr4b* and *brn3a* appears unaffected in *neurod4* morpholino injected embryos (C, n = 13/13 and G, n = 14/15) as compared with the expression in wild type (A, n = 9/9 and E, n = 9/9); *cxcr4b* and *brn3a* expression is also unaffected in *neurog1*^*hi1059*^ mutant (B, n = 12/14 and F, n = 9/11) or only slightly decreased in few cases (respectively n = 2/14 and n = 2/11; data not shown). In contrast, the expression of both genes is either abrogated or strongly reduced in *neurog1*^*hi1059*^;*neurod4* morphants (D, n = 16/16 and H, n = 12/14). Embryos are viewed dorsally with anterior up.

Proneural genes of the atonal and achaete-scute families encode bHLH transcription factors that are key regulators of neurogenesis in both vertebrates and invertebrates. The zebrafish atonal homologue *neurogenin1* (*neurog1*) is expressed in habenular progenitors [25,40]. To address whether Neurog1 controls habenular neurogenesis we examined the expression of well-described habenular markers in embryos homozygous for the *neurog1*^*hi1059*^ mutant allele [28]. Expression of the chemokine receptor *cxcr4b*, a marker of habenular progenitors [25], is largely unaffected in the absence of Neurog1 function (Fig 1A and 1B). Likewise, no differences were detected between wild type and *neurog1*^*hi1059*^ mutant siblings in the expression of the homeo-domain transcription factor *brn3a*, a marker of post-mitotic habenular neurons [17,25] (Fig 1E and 1F). Thus, as previously reported by Kuan at al, loss of Neurog1 function alone does not affect habenular development [41]. During development of the zebrafish cranial ganglia and olfactory system, Neurog1 acts redundantly with a second atonal-like proneural factor, Neurod4 [38,42]. To address if a similar situation exists during habenular neurogenesis, we injected *neurod4* specific morpholinos into embryos from crosses between heterozygous *neurog1*^*hi1059*^ mutant carriers and examined the effect of the loss-of-function of both bHLH factors on the expression of habenular markers. As for the simple *neurog1* mutant loss-of-function context, knock-down of Neurod4 alone had no effect on the expression of *cxcr4b* or *brn3a* (Fig 1C and 1G). In contrast, the expression of *cxcr4b* and *brn3a* was respectively absent or strongly reduced in embryos lacking the activity of both *neurog1* and *neurod4* (Fig 1D and 1H). We conclude that the redundant proneural activity of *neurog1* and *neurod4* is required for habenular neurogenesis.

### Habenular neurogenesis requires Pax6a

**Fig 2:**
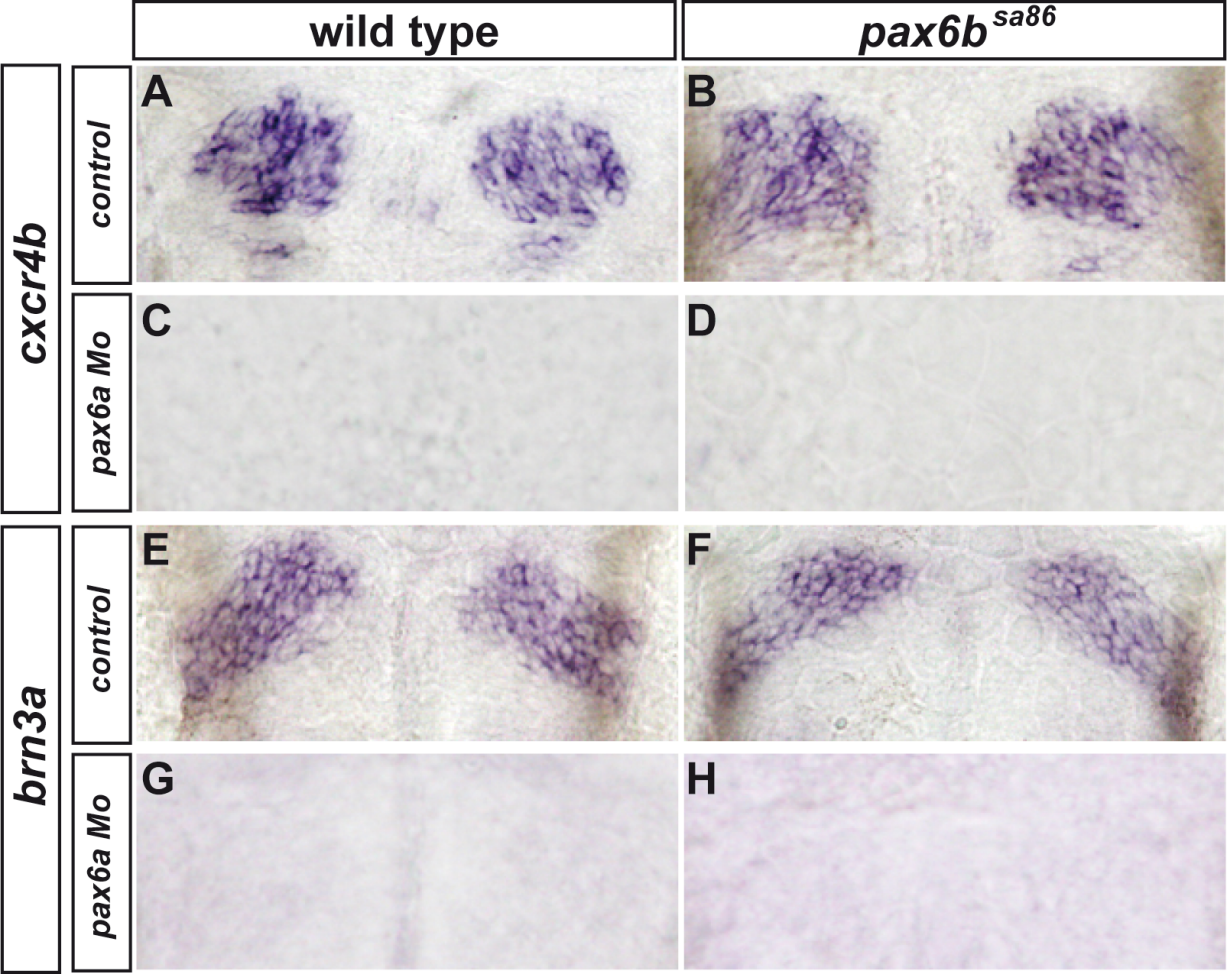
Pax6a but not Pax6b are required for habenular neurogenesis. Whole-mount *in situ* hybridization against *cxcr4b* (A-D) or *brn3a* (E-H) showing the epithalamus of wild type (A,E), *pax6b*^*sa86*^ mutant embryos (B,F), *pax6a* morpholino injected embryos (C,G) or *pax6b*^*sa8*^;*pax6a* morphant embryos (D,H) at 36 (A-D) or 48 (E-H) hpf. Whereas the expression of *cxcr4b* and *brn3a* appears unaffected in *pax6b*^*sa86*^ mutant embryos (B, n = 6/6 and F, n = 5/5) compared to the expression in wild type controls (A and E, n = 5/5), expression of both genes is abrogated after injection of morpholinos against *pax6a* in either wild type (C, n = 4/6 and G, n = 6/8) or *pax6b*^*sa8*^ embryos (D and H, n = 4/4). Embryos are viewed dorsally with anterior up.

Pax6 encodes a paired homeodomain transcription factor that acts in a concentration-dependent manner during region-specific differentiation of neural tissues [43]. The two zebrafish *pax6* orthologues, *pax6a* and *pax6b*, are expressed in large, overlapping domains in the diencephalon [36,44] and have been implicated in aspects of diencephalon development [45]. We have previously shown that the zebrafish *pax6* genes directly regulate *neurog1* expression in the zebrafish diencephalon via a conserved cis-regulatory module located immediately upstream of the *neurog1* coding region [46]; a similar regulatory paradigm exists between Pax6 and Neurog2 in the mouse telencephalon and spinal cord [47]. However, while simultaneous knock-down of the two *pax6* orthologues affects the expression of *neurog1*, the consequences on habenular neurogenesis are not known in zebrafish [46]. To address this question, we injected *pax6a* morpholinos into embryos from crosses between heterozygous *pax6b*^*sa86*^ mutant carriers and examined the expression of early and late habenular markers. Homozygous *pax6b*^*sa86*^ mutant embryos display *cxcr4b* and *brn3a* expression in the habenulae largely indistinguishable from that of wild type siblings (Fig 2A, 2B, 2E and 2F). Somewhat surprisingly, on the other hand, morpholino knock-down of *pax6a* alone abrogated the expression of both markers (Fig 2C and 2G); *pax6a* morphant/*pax6b*^*sa86*^ mutant embryos behaved the same as *pax6a* morphants alone with respect to these markers (Fig 2D and 2H). These results suggest that while both *pax* genes appear to be required for the expression of *neurog1* widely throughout the diencephalon, Pax6a but not Pax6b is needed for the expression domain of *neurog1* driving habenular neurogenesis. A more precise analysis in the epithalamus shows that *pax6a* expression overlaps extensively with GFP from a *Tg(neurog1*:*GFP)* transgene prior to the appearance of habenular neurons (S1 Fig). Together with our previous work, this suggests that Pax6a regulation of *neurog1* in this domain is direct [46].

**Fig 3:**
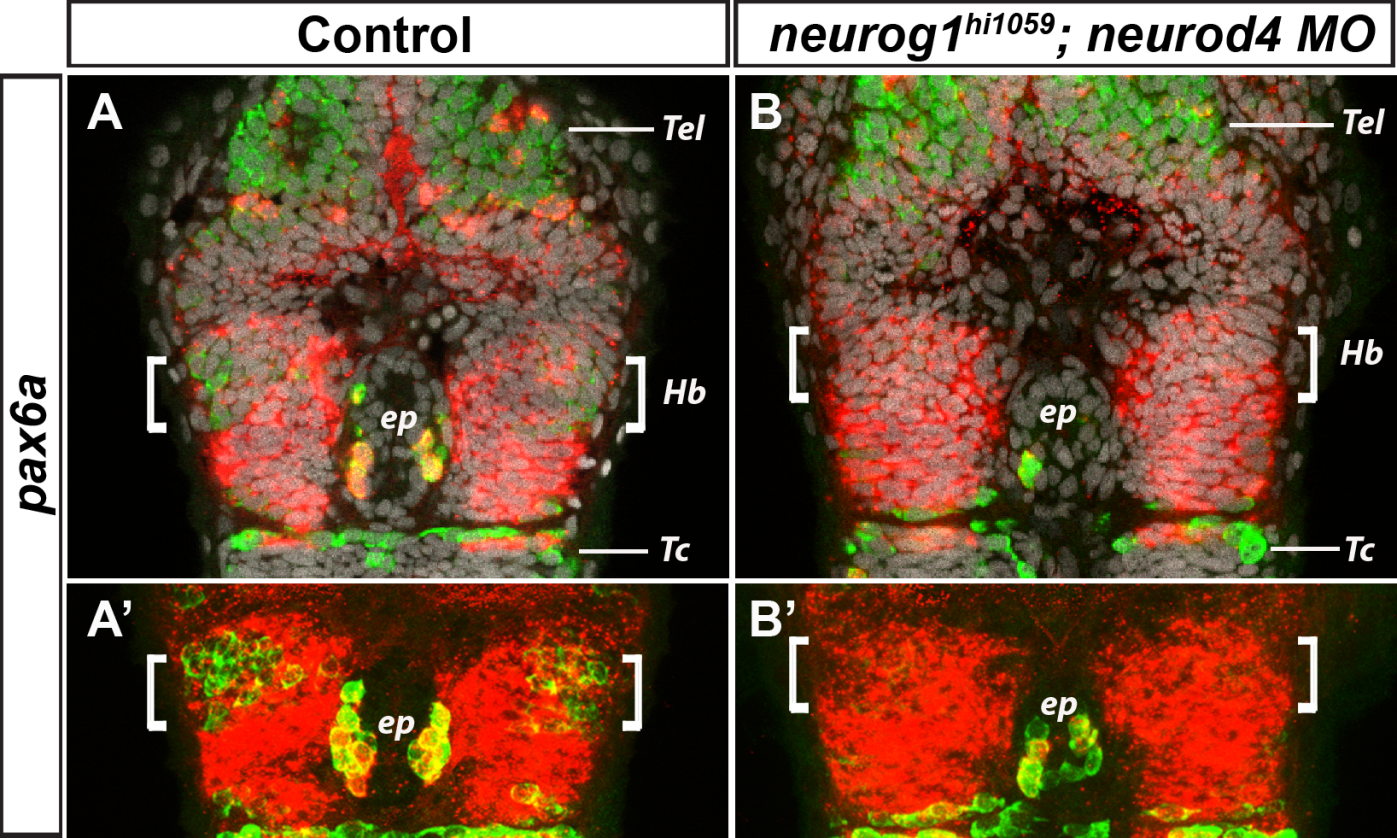
Expression of *pax6a* is unaffected in *neurog1*^*hi1059*^*;neurod4* morphant embryos. Confocal sections (A,B) or 10μm maximum projections (A’,B’) of the head of wild type (A; n = 6/6) or *neurog1*^*hi1059*^;*neurod4* Mo injected embryos (B; n = 10/10) at 36 hpf after a whole-mount *in situ* hybridization against *pax6a* (red) and an immunostaining against HuC/D protein (green); cell nuclei staining (in grey) makes visible brain structures (A,B). As previously described, the neuronal marker HuC/D is expressed in the epithalamus, both in epiphyseal projection neurons (ep) and in habenular neurons (Hb, white brackets) located on either sides of the epiphysis. The expression of HuC/D is abrogated or strongly reduced in the habenular domain of *neurog1*^*hi1059*^;*neurod4* morphant embryos, while it is still detected in the telencephalon (Tel), in the epiphysis and in neurons of the tectum (Tc) (B,B’). On the contrary, no change is seen in the expression of *pax6a* in the same region, suggesting that the expression of *neurog1* and *neurod4* does not regulate *pax6a* during habenular neurogenesis. Embryos are viewed dorsally with anterior up. The expression of HuC/D and *pax6a* in *neurog1*^*hi1059*^ mutants (n = 5/5) and *neurod4* Mo injected embryos (n = 11/11) was similar to that observed in the non-injected controls (data not shown).

Scardigli and colleagues reported that while Pax6 directly regulates Neurog2 expression in the mouse spinal cord, Neurog2 feeds back onto Pax6 to maintain its expression [47,48]. To address whether similar feedback control is active during habenular neurogenesis in zebrafish, we assayed the expression of *pax6a* in *neurog1*^*hi1059*^;*neurod4* morphant embryos. As shown in Fig 3, no change in the expression of *pax6a* was detected in the epithalamus of the *neurog1*^*hi1059*^;*neurod4* morphant embryos (Fig 3A and 3A’ versus 3B and 3B’). To address whether habenular neurogenesis was affected in this double loss of function context, we analysed the expression of HuC/D in the epithalamus of the same *neurog1*^*hi1059*^;*neurod4* morphant embryos; the RNA-binding protein HuC is a general marker of newly differentiated neurons that is expressed in the epithalamus in both habenular neurons and epiphysial projection neurons [25,49]. HuC/D was not detected at 36 hpf in the habenulae of *neurog1*^*hi1059*^;*neurod4* morphant embryos, confirming a requirement of *Neurog1 and Neurod4* for habenular neurogenesis (Fig 3A–3B’). Thus, our results suggest that there is no feedback control of *neurog1* and *neurod4* on *pax6a* in this system.

### Hedgehog signalling is required for habenular neurogenesis

**Fig 4:**
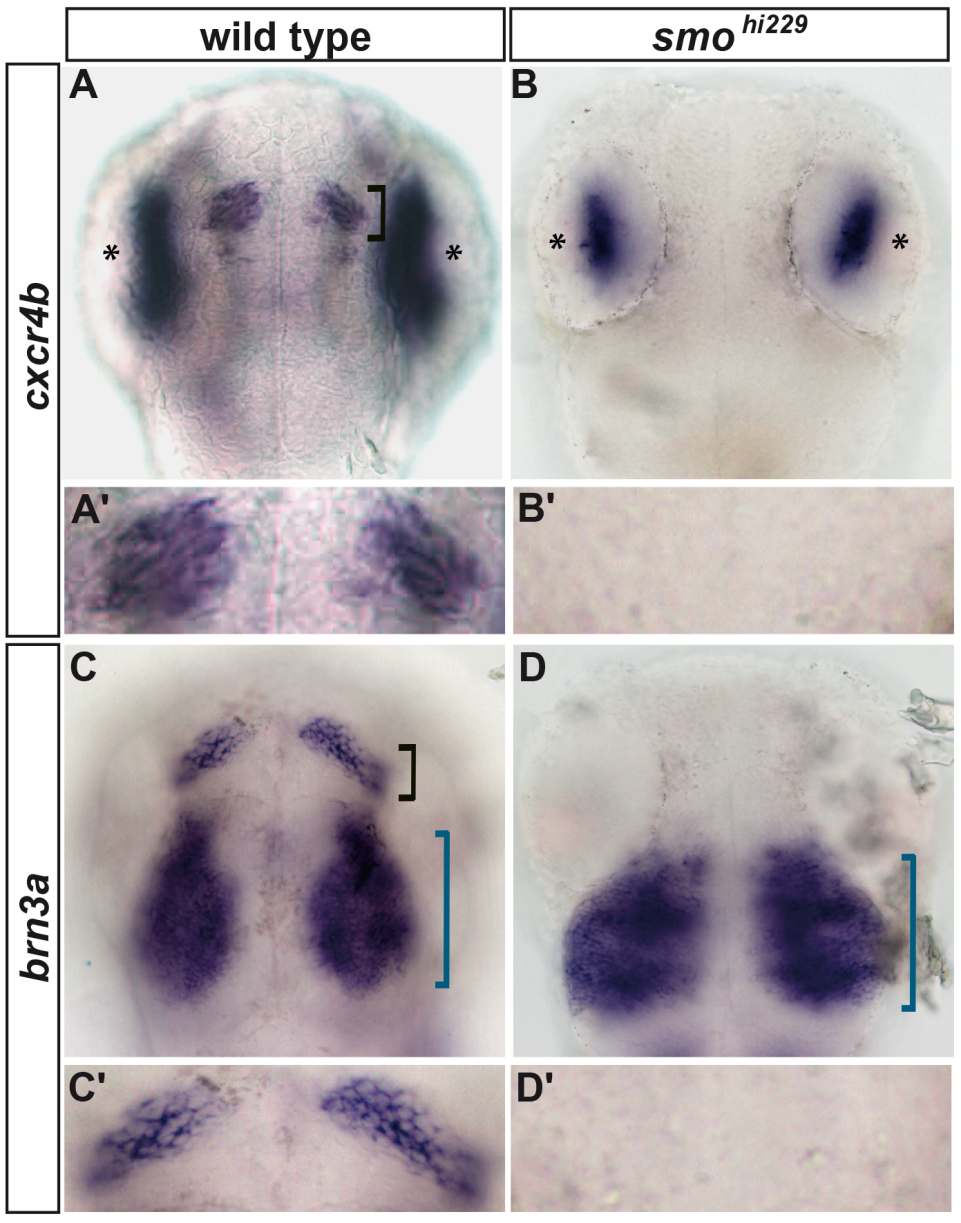
Habenular neurogenesis requires hedgehog signalling. Whole-mount *in situ* hybridization against *cxcr4b* at 36 hpf (A-B’) or *brn3a at* 48 hpf (C-D’) showing heads (A-D) or the epithalamus (A’-D’) of wild type (A,A’,C,C’) or *smo*^*hi229*^ embryos (B,B’,D,D’). The expression of *cxcr4b* and *brn3a* is detected in the habenulae of all the wild type siblings (n = 21/21 and n = 6/6, respectively). While residual expression of *cxcr4b* in the eyes (*) and *brn3a* in the tectum (blue bracket) is visible in *smo*^*hi229*^ embryos, the expression of both genes is abrogated in the habenular nuclei (Black bracket, B,B’, n = 12/13 and D,D’, n = 6/6). Embryos are viewed dorsally with anterior up.

Next, we screened existing genetic and pharmacological tools to identify signalling cascades that might regulate habenular neurogenesis upstream of the Pax6a>Neurog1/Neurod4 cassette. Interestingly, whereas the *cxcr4b* is robustly expressed in habenular progenitors of wild type embryos at 36 hours post-fertilisation (hpf) (Fig 4A and 4A’), *cxcr4b* expression is absent in the epithalamus of embryos mutant for *smoothened*, a gene encoding a non-classical G protein-coupled receptor required for Hedgehog (Hh) signalling (*smo*^*hi229*^; [28]; Fig 4B and 4B’); residual expression of *cxcr4b* in retinal ganglion cells of *smo*^*hi229*^ mutant embryos suggests that the lack of expression in the habenulae is due to a lack of habenular progenitors and not because *cxcr4b* is a direct target of Hh signalling. Similarly, the expression of *brn3a* is absent from the epithalamus of *smo*^*hi229*^ mutant embryos at 48 hpf but is still expressed in the developing tectum (Fig 4C–4D’).

To further support our data showing a requirement for Hedgehog signalling in habenular neurogenesis, we analysed the expression of the habenular markers *cxcr4b* and *brn3a* in embryos mutant for sonic hedgehog (*shha*^*tbx392*^/*sonic-you)*; *shha* is expressed in the Zona Limitans Intrathelamica (ZLI) where it is required for the regionalisation of the Thalamus [50,51]. Although the phenotype obtained in *shha*^*tbx392*^ mutant embryos is not as severe as that observed in *smo*^*hi229*^ mutant embryos, we found that the expression of both *cxcr4b* and *brn3a* are strongly reduced in this context. These data suggest that Shha is the main Hedgehog ligand involved in habenular neurogenesis (S2 Fig).

**Fig 5:**
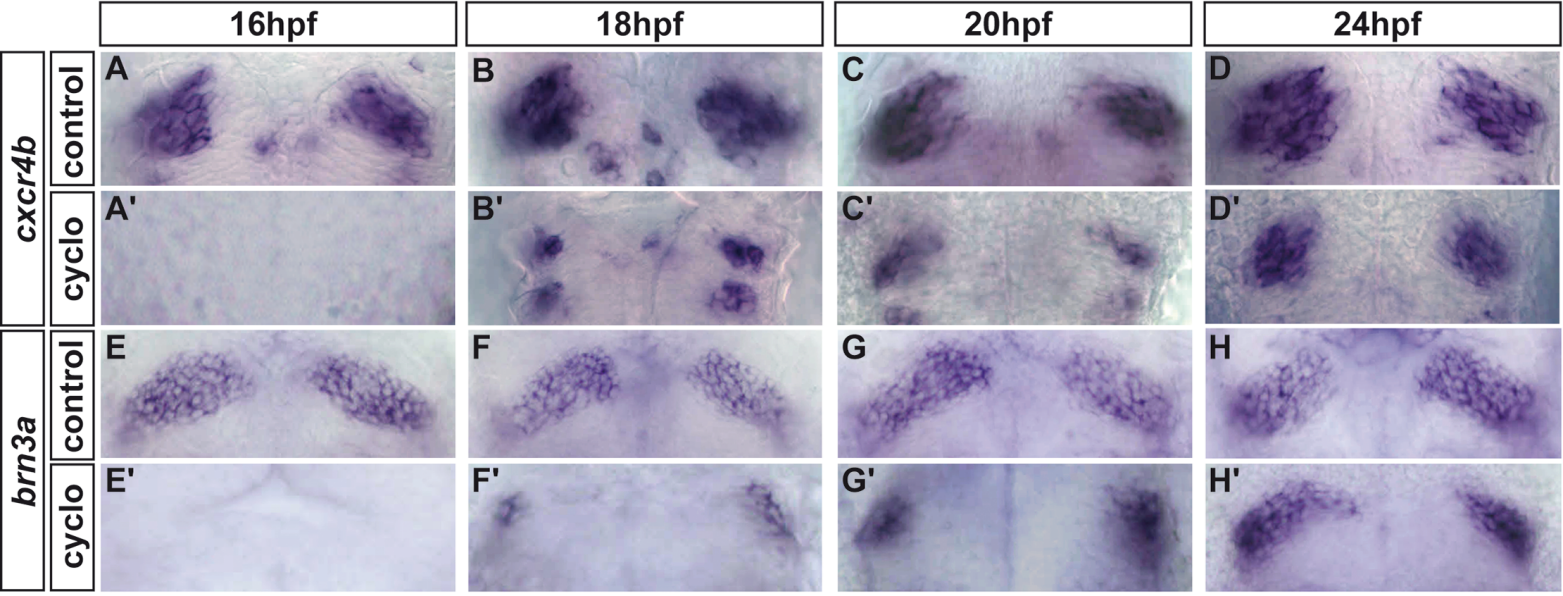
Hedgehog signalling is required continuously from 16hpf for correct habenular development. Whole-mount *in situ* hybridization against *cxcr4b* (A-D’) or *brn3a* (E-H’) showing the epithalamus at 36 or 48 hpf, respectively, in control treated (A-D, E-H) or cyclopamine treated embryos from various stages (A’-D’, E’-H’). Cyclopamine treatment from 16 hpf completely abolishes the expression of *cxcr4b* (n = 19/21) and *brn3a* (n = 22/22) in habenular progenitors and neurons respectively. Treatment from 18 hpf results in either a similar abrogation of *cxcr4b* and *brn3a* expression (respectively n = 6/11 and n = 6/8) or in a strong decrease (n = 5/11 and n = 2/8, representative pictures in B’ and F’). The expression of *cxcr4b* and *brn3a* recovers if embryos are treated at progressively later stages. Although their expression level is significantly reduced, *cxcr4b* and *brn3a* are expressed in the habenulae of about half of the embryos treated with cyclopamine from 20 hpf (respectively C’, n = 20/37 and G’, n = 12/22) and in most of the embryos treated from 24 hpf (D’, n = 37/42 and H’, n = 28/34); in the remaining embryos, the expression was not detected (C’, n = 17/37, G’, n = 10/22, D’, n = 5/42 and H’, n = 6/34). For ethanol treated control embryos, the following numbers of embryos were examined: n = 19 (A), n = 3 (B), n = 10 (C), n = 9 (D), n = 19 (E), n = 5 (F), n = 8 (G), n = 7 (H). Embryos are viewed dorsally with anterior up.

**Fig 6:**
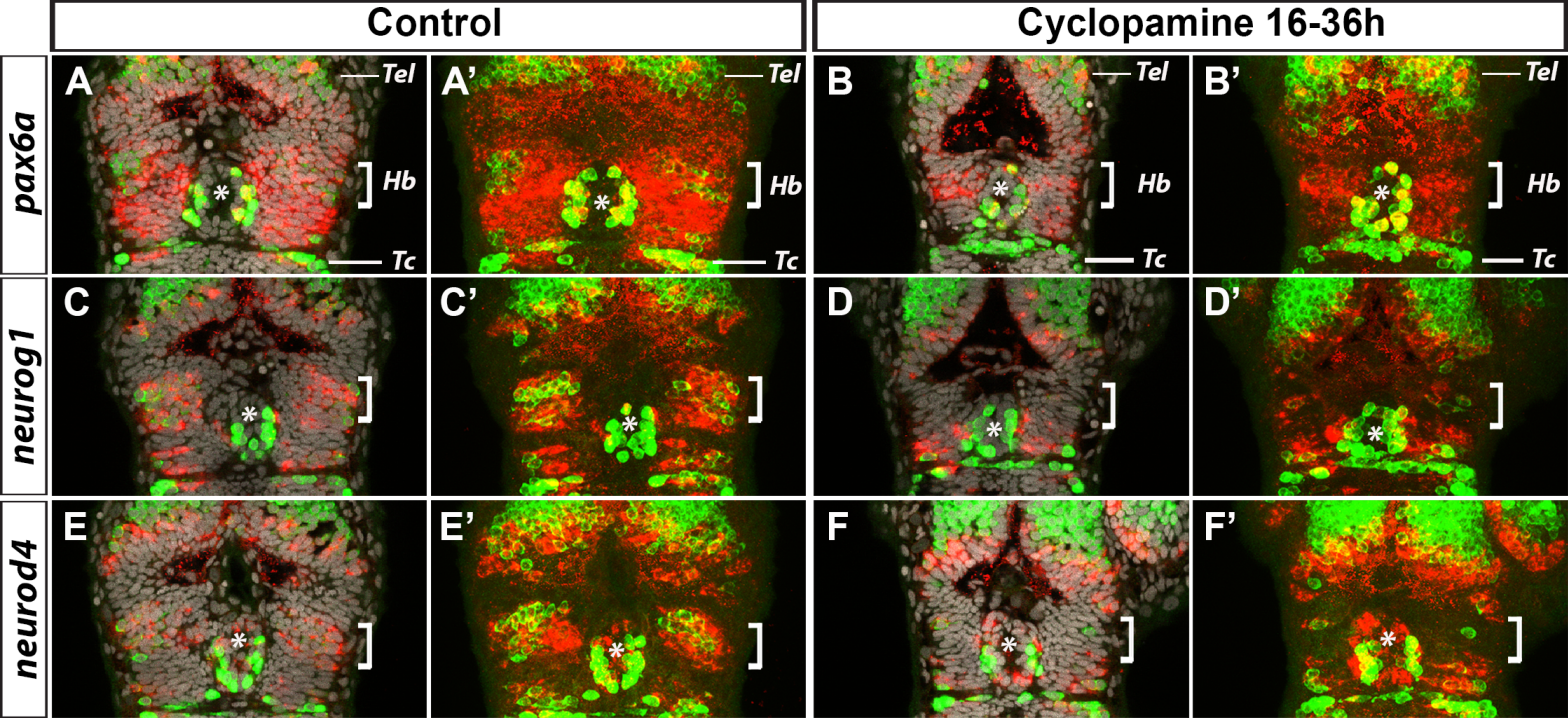
Hh signalling is required for the expression of *pax6a*, *neurog1* and *neurod4* in the Habenular nuclei. Confocal sections (A-F) or 15μm maximum projections (A’-F’) showing the heads of control treated embryos (A-A’, n = 9; C-C’, n = 6; E-E’, n = 6) or those treated from 16 hpf with cyclopamine (B-B’, n = 10, D-D’, n = 7, F-F’, n = 6) after a whole-mount *in situ* hybridization against *pax6a* (A,A’,B,B’), *neurog1* (C,C’,D,D’) and *neurod4* (E,E’,F,F’) (red) and immunostaining against HuC/D protein (green); cell nuclei staining (grey) makes visible brain structures in the confocal sections (A-F). The overall morphology of the head appears normal in cyclopamine treated embryos and the expression of HuC/D does not appear to be affected in the telencephalon (Tel), in the epiphysis (*) nor in the tectum (Tc). In contrast, HuC/D expression is absent or strongly reduced in the habenular domain (Hb, white brackets) of cyclopamine treated embryos. The expression of *pax6a* is strongly reduced (B-B’, n = 10/10) in the habenular domain (Hb, white brackets) of cyclopamine treated embryos. The expression of *neurog1* and *neurod4* is also abrogated specifically in the habenular domain of cyclopamine treated embryos (D-D’, n = 7/7, F-F’, n = 6/6). All embryos are at 36 hpf. Embryos are viewed dorsally with anterior up.

Birthdating experiments have shown that habenular neurons begin to leave the cell cycle around the end of the first day of development [24]. However, it is not clear from our results with *smo*^*hi229*^ and *shha*^*tbx392*^ mutant embryos at which stage the Hh pathway is required for habenular neurogenesis. To determine the time window during which Hh signalling is required, we used cyclopamine to inhibit Hh signalling starting at various developmental stages up to 24 hpf [52,53]. Embryos treated with ethanol from 16 hpf display robust expression of *cxcr4b* and *brn3a* in the habenulae at 36 and 48 hpf, respectively (Fig 5A and 5E). Conversely, cyclopamine treatment from the same stage abolishes the expression of both markers (Fig 5A’ and 5E’); HuC/D labelling was also lost in the habenulae of embryos treated with cyclopamine from 16 hpf but not affected in the epiphysis, in the telencephalon or in neurons in the tectum (Fig 6B–6F’). Blocking Hh signalling from 18 hpf still profoundly affects the habenular expression of *cxcr4b* and *brn3a*, although the effect is not as penetrant as upon treatment at the earlier stage with residual expression of the two markers being detected in some embryos (Fig 5B and 5F versus 5B’ and 5F’). Blocking Hh signalling with cyclopamine from 20 hpf and 24 hpf has a far less pronounced effect but still results in a decrease in the expression of *cxcr4b* or *brn3a* in the epithalamus (Fig 5C and 5G versus 5C’ and 5G’ and 5D and 5H versus 5D’ and 5H’). Taken together, our results indicate that Hh signalling acts as an obligate regulator of habenular neurogenesis and that the pathway is required continuously starting from between 16–18 hpf until at least 24 hpf for the production of habenular neurons.

To determine if Hh signalling lies upstream of the Pax6a>Neurog1/Neurod4 cassette we have described above, embryos were treated with cyclopamine from 16 hpf and the expression of *pax6a*, *neurog1* and *neurod4* was analysed by in situ hybridisation. Comparing lateral views of control and cyclopamine treated embryos shows that the expression of *pax6a*, *neurog1* and *neurod4* appear specifically affected in the dorsal part of the diencephalon (S3 Fig). To understand better the changes induced by cyclopamine treatment, we analysed the expression of these three same genes (*pax6a*, *neurog1* and *neurod4*) together with the expression of HuC/D protein at the level of the epithalamus by confocal imaging. As shown in Fig 6, while the expression of *pax6a* is globally similar between control and cyclopamine treated embryos in the telencephalon or the epiphysis, expression in the habenular territory is strongly reduced when Hh signalling is abrogated (Fig 6A and 6A’ versus 6B and 6B’). Staining for cell nuclei indicate that the size of the prospective habenulae is significantly decreased in cyclopamine treated embryos suggesting that the loss of *pax6a* expression in the dorsal epithalamus is partly due to reduced tissue size (Fig 6A–6F). Nonetheless, the residual habenular progenitors also display decreased *pax6a* expression. Similarly, the expression of the two bHLH proneural genes, *neurog1* and *neurod4*, is also severely affected in the epithalamus (Fig 6C–6F versus 6C’–6F’). Together these results suggest that a Hh, Pax6a, Neurog1/Neurod4 cascade is required for habenular neurogenesis.

### Hedgehog signalling is required cell autonomously for habenular neurogenesis

**Fig 7:**
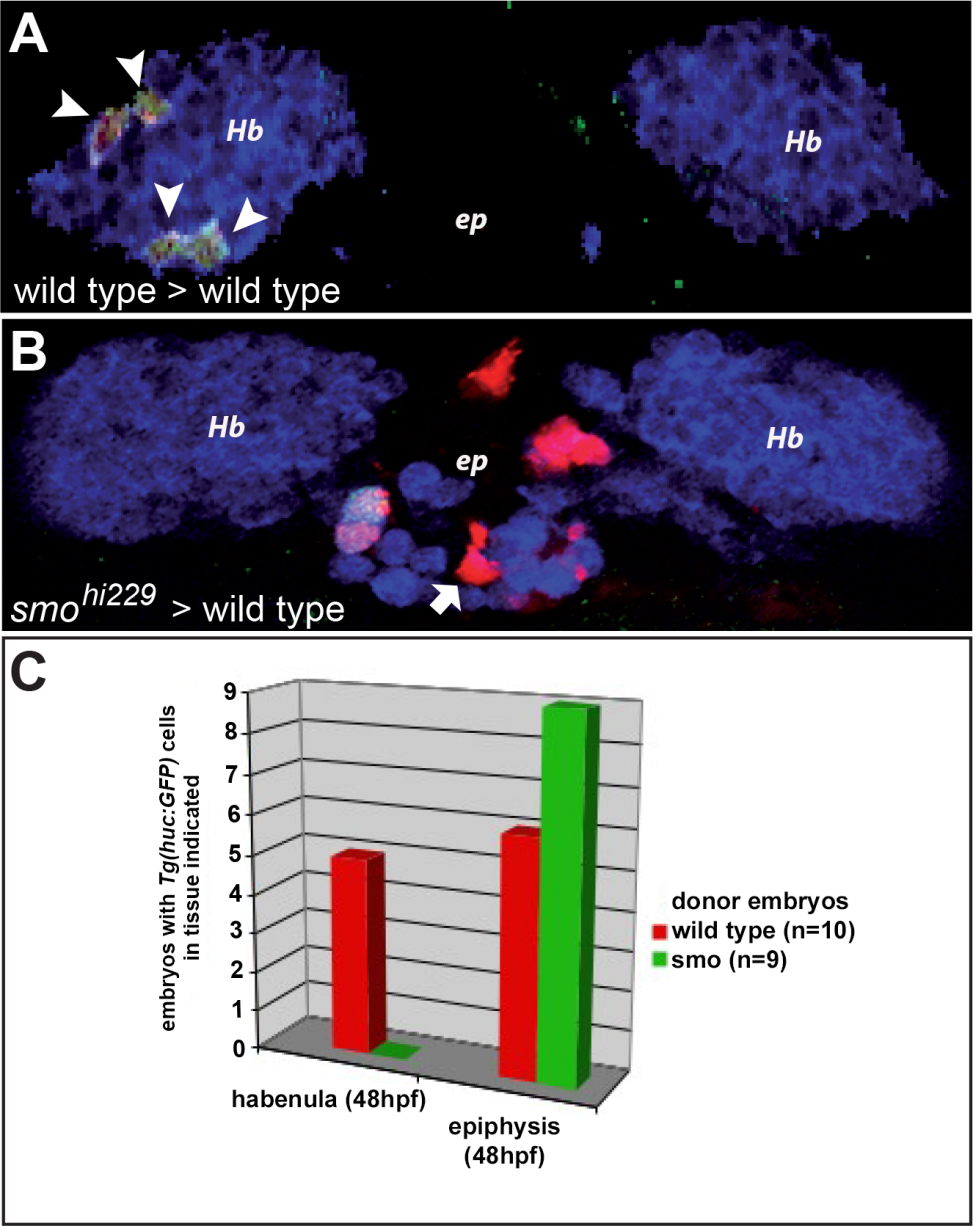
Autonomous reception of Hedgehog signals is required for habenular neurogenesis. Confocal sections through the epithalamus of wild type host embryos containing transplanted cells from wild type (A) or *smo*^*hi229*^ donors (B). Donors carried the *Tg(huC*:*GFP)*, which appears in white, and transplanted cells appear in red; red cells that are not white in B are epiphysial photoreceptors (white arrow). Host embryos were immunolabelled with an anti-HuC/D antibody (in blue) that highlights the habenular neurons (Hb) and projection neurons in the epiphysis (ep). Embryos are viewed dorsally with anterior up. A histogram indicates the number of embryos incorporating cells from wild type or *smo*^*hi229*^ mutant donors into either the habenulae or epiphysis (C). Unlike cells transplanted from wild type donors (white arrowheads in A), those from homozygous *smo*^*hi229*^ mutants are systematically excluded from becoming habenular neurons; transplanted cells from either donor can become epiphysial projection neurons.

The marker studies presented here indicate that Hh signalling is required for the generation of habenular progenitors and post-mitotic habenular neurons. However, it is not clear if the reception of Hh is autonomously required in this process. To address this question we created mosaic embryos containing clones of cells unable to respond to Hh signalling. For this, cells from rhodamine-dextran loaded embryos produced by crosses between identified *smo*^*hi229*^ heterozygotes carrying the *Tg(huC*:*GFP)* were transplanted into wild type hosts; the transgene *Tg(huC*:*GFP)* recapitulates the expression of the endogenous *huC* gene in the epithalamus [25,31]. Both donor and host embryos were then grown to 48 hpf at which stage donors were genotyped and hosts were fixed for analysis. As expected, cells transplanted from wild type or *smo*^*hi229*^ heterozygous embryos were able to integrate into the epithalamus, being found in either the habenulae or epiphysis with an approximately equal frequency (Fig 7A and 7C). Furthermore, transplanted cells detected in the habenula expressed GFP from the *Tg(huC*:*GFP)* transgene indicating that they had become post-mitotic habenular neurons (Fig 7A). Cells incorporated into the epiphysis also expressed GFP if they differentiated as projection neurons but remained GFP-negative if they developed as photoreceptors (data not shown; [49]). Conversely, while cells transplanted from homozygous *smo*^*hi229*^ mutant embryos were capable of becoming epiphysial projection neurons, they were systematically excluded from becoming habenular neurons (Fig 7B and 7C). We conclude that while Hh signalling does not appear to be required for projection neuron development in the epiphysis, activation of the Hh pathway is required in an autonomous fashion for correct habenular neurogenesis.

## Discussion

Habenular nuclei act as a major relay component of a conserved conduction system between the forebrain and midbrain, and have been implicated in modulating different behaviours in a variety of animal models. However, in contrast to others structures in the diencephalon, the development of the habenulae and the mechanisms involved in the generation and differentiation of habenular neural progenitors have not been studied in detail. Here, we present the characterisation of a cassette of transcription factors, Pax6a/Neurog1/NeuroD4, that is required for habenular neurogenesis. We show that Hedgehog (Hh) signalling is required upstream of the expression of Pax6a, which is itself necessary for the expression of a pair of proneural bHLH genes. Pharmacological inhibition of the Hh pathway from 16 hpf results in the complete loss of habenular neurons, and our data show that Hh signalling is required continuously at least until 24h for the specification of dorsal habenular neuronal progenitors. Finally, cell-transplantation experiments indicate that Hh is required autonomously for the generation of habenular neurons.

### Hh signalling in the development of the diencephalon

The developing diencephalon is composed of three so-called prosomeres that give rise to the prethalamus rostrally (p3), the thalamus and epithalamus (p2) and the pretectum (p1) caudally [54]. Between the p3 and p2 region is a transverse domain, the *zona limitans intrathalamica* (ZLI), that expresses Hh signals and acts as an organiser for diencephalon development and regionalisation (for review, see [55,56]). Hedgehog signalling from this region promotes the robust expression of regionally specific genes on both sides of the ZLI that establish the molecular identity of both the pre-patterned prethalamus and thalamus [50,51]. In contrast to its well-documented role in prethalamus and thalamus, the function of Hh signalling in the development of the most dorsal part of the p2 prosomere, the epithalamus, has been largely neglected.

In a study that addressed the role of Sonic hedghog (Shh) in the mouse thalamus, no significant defects in habenular morphology were reported in *Shh* mutants (Fig 4 in [57]). Similarly, Vue and colleagues did not report any alterations in the epithalamus after reduction of Hh signalling although they found that increased Hh signalling in the diencephalon induces an expansion of markers for the thalamic progenitor domain at the expense of some markers for the pretectum and habenula [58]. More recently, two studies proposed that enhanced Shh signalling in the ZLI might account for the diencephalic defects observed in *Pax6* mutant embryos [45,59]. Chatterjee and colleagues reported an increase in the expression of the pineal marker *otx5* in the absence of Hh signalling in zebrafish embryos [45]. The authors also analysed the expression of *cxcr4b* in the epithalamus but it is not clear, however, whether the changes detected concern the habenulae or the epiphysis (Fig 8 in [45]) as this gene is also expressed in epiphysial projection neurons at 28 hpf (S4 Fig). Altogether, our results from markers analysis and transplants experiments indicate that Hedgehog signalling is absolutely required in zebrafish for the development of the dorsal habenular nuclei but not for the specification of the epiphysis. Thus, Hh signalling seems to have differential roles within the epithalamus on the habenular and the epiphysis.

Previous studies have suggested that Smoothened mediates Hh signalling from all zebrafish Hh signals [60]. A study showed that Shha/Sonic Hedgehog and Shhb/Twhh (Tiggy-winkle hedgehog) act redundantly during the specification of prethalamus identity while the induction of the thalamus seems to rely only on Shha [51]. Although *cxcr4b* and *brn3a* expression are strongly reduced in *shha*^*tbx392*^ mutants, we found that both markers are still detected in this context, a phenotype weaker than that observed in *smo*^*hi229*^ mutant in which *cxcr4b* and *brn3a* expression are fully abolished in the habenulae. This result suggests that the requirement for hedgehog signalling during habenular neurogenesis mainly relies on Shha but might also involve the partially redundant activity of Shhb.

Our results from cyclopamine treatment suggest that the Hh pathway acts early in the development of the habenulae. Indeed, cyclopamine treatment interferes with the production of the earliest habenular progenitors/neurons only if it is performed starting at 16 hpf; *brn3*+ neurons and *cxcr4b*+ progenitors are detected in only a few embryos treated from 18 hpf and in about half of embryos treated from 20 hpf. We previously described that the first HuC+ habenular neurons appear at 30 hpf and express *cxcr4b* from 28 hpf [25]. Thus, Hh signalling is required about 12 hours before the appearance of the first neurons in the dorsal habenula. From this result, one could suggest that Hh signalling plays an early role in patterning the epithalamus, being required to specify the territory from which habenular progenitors will emerge, for instance. The observed decrease in the global size of prospective habenular domain upon cyclopamine treatment (Fig 6) might support this hypothesis. However, the fact that Hh signalling is required continuously for the generation of habenular neurons suggests that Hh signalling is not only acting to pattern the diencephalon.

### Hh signalling and Pax6 cross-regulation in the development of the diencephalon

The homeobox transcription factor Pax6 is expressed in a highly dynamic pattern in the anterior neural plate during development and this is consistent with a suggested requirement for this gene in different step of forebrain development [61]. Early studies in the mouse reported that Pax6 is required for normal development and regionalisation of the diencephalon [62,63]. Our previous work suggested that zebrafish Pax6a and Pax6b could act as pre-pattern genes in the diencephalon by defining a large domain in which other factors, such as the proneural factor Neurog1, would further specify sub-domains of neurogenesis [46]. Recently, mouse Pax6 has been shown to control the patterning of the diencephalon through the cell autonomous repression of *Shh* expression in the ZLI [59]. Similarly, Chatterjee et al showed that Pax6 positively regulates the size of the epithalamus through a spatio-temporal control of *Shh* expression in both mouse and zebrafish [45]; in the latter model, this function apparently involves the redundant action of both Pax6a and Pax6b. In the present study, we show that only one of the two zebrafish *pax6* genes, *pax6a*, is required for habenular neurogenesis. Thus, although Pax6a and Pax6b are redundantly required upstream of Shh to restrict its expression in the ZLI, only Pax6a appears to be required downstream of Hh signalling to activate the expression of the proneural genes *neurog1* and *neuroD4*. These results suggest that Pax6 is reiteratively used during habenular development and that the duplicated *pax6* genes in zebrafish have acquired specific function.

Whether Hh signalling positively regulates *pax6a* expression directly is difficult to address; our results only suggest that Hh signalling acts positively upstream of Pax6a expression. This contrasts with the negative control of Pax6 by Shh described in other developmental systems. For instance, Shh has been shown to negatively regulate *Pax6* expression in the spinal cord [64]. In the chick diencephalon, however, Shh has been shown to control Pax6 expression in opposite ways on either side of the ZLI, as it is required for the maintenance of expression in the prethalamus but inhibits expression in the thalamus [50].

### Links between Hedgehog and other signalling pathways during habenular neurogenesis

Whether Hh signalling interacts with other pathways known to be involved in habenular development is an open question. For instance, it has been shown that Fgf8 is required for the development of the habenulae in both mouse and zebrafish [65,66]. Furthermore, previous work has suggested cross-regulation between the Fgf and Shh pathways in mouse [67] and zebrafish [68]; in contrast, Martinez-Ferre et al. found no alteration in *SHH* expression in the ZLI of *FGF8* mouse mutants [65]. Wnt signalling has also recently been implicated in the development of the dorsal habenular nuclei [41,69]. Mutations in *wntless*, a conserved gene involved in the transport of Wnt ligands, lead to a strong reduction in the size of the dorsal habenulae [41]. This defect correlates with a reduction in the epithalamic expression of *neurog1* and in the pool of *cxcr4b* expressing habenular progenitors; the effect of the *wls* mutation on the expression of *pax6a* is subtle at best, suggesting that Wls acts downstream of or in parallel with Pax6a to control *neurog1* expression [41]. Interestingly, no change in the expression of several targets of Hedgehog signalling (*oligo2* and *nkx2*.*2*) or *fgf8* is detected in *wls* mutants, suggesting that these pathways lie upstream of Wnt signalling in this context. Further investigation will be necessary to address specifically how the Hedgehog, Fgf and Wntless-dependant Wnt pathways act during habenular development.

### Asymmetric habenular neurogenesis

The habenulae have emerged as a model to understand how left-right (LR) asymmetry develops in the brain. We have previously shown that Nodal signalling biases the timing of habenular neurogenesis so that habenular progenitors and early born neurons appear initially on the left [25]. The functional relevance of the asymmetry in the pool of early born habenular neurons remains unclear as robust asymmetry of molecular markers and connectivity is still detected in the habenulae when Nodal is absent or bilateral, although the orientation of the asymmetry is randomised [70,71]. Nonetheless, this Nodal-dependant asymmetry in neurogenesis might have a subtle and as yet unrecognised impact on habenular asymmetry. For instance, the early asymmetric neurogenesis could partly contribute to the residual asymmetry in habenular projection to the IPN that remains after parapineal ablation in zebrafish embryos [18,19]. A recent study suggests that Nodal mediated asymmetric neurogenesis in zebrafish could be a vestige of an ancestral role of Nodal in driving habenular asymmetry in early vertebrates [72]. Indeed, in the catshark and lamprey, two species that either lack a parapineal or display a non-lateralised parapineal respectively, habenular asymmetry is conserved and develops solely through a Nodal-dependant mechanism. Thus, understanding how Nodal drives asymmetry in the timing of habenular neurogenesis in zebrafish is important as it might reveal mechanisms by which habenular asymmetry is established in early vertebrates.

How the Nodal pathway imposes an asymmetry in the timing of habenular neurogenesis is not known yet. In the light of our results, we can speculate that this function of Nodal could involve modulation of Hh signalling. For instance, it is known that in the neural tube both the concentration and the duration of Hh signalling can be integrated by neural progenitors to promote different neural fates [73]. It is possible, therefore, that left-sided Nodal signalling in the epithalamus might result in progenitors of the left and right habenular neurons being exposed to slightly different concentration of Hh signals or for a different length of time, which could trigger a subtle heterochrony in their differentiation. Addressing this possibility will require investigating left and right differences in expression of endogenous read-outs or reporter transgenes for the Hh pathway over time. Alternatively, Nodal could indirectly affect the response of left habenular progenitors to Hh signals through the modulation of others signalling pathway. One such candidate is the Notch pathway that is known to potentiate Hh responsiveness in neural progenitors [74,75]. Other putative targets of the Nodal pathway could be the Fgf or Wntless-dependant Wnt pathways as these have also been implicated in the development of the dorsal habenulae, as mentioned above. A better understanding of which epithalamic cells receive Nodal signals and what are the Nodal pathway targets genes involved should help address how Nodal signalling biases the genetic programme of early neurogenesis that we describe here.

### Ventral versus dorsal habenula

The habenular nuclei are composed of a ventral and a dorsal domain that are homologous to the medial and lateral sub-domains of mammals [16]. In our study, we show a requirement for Hh signalling upstream of a Pax6/Neurog1/NeuroD4 cassette for the generation of neural progenitors of the dorsal habenular. It remains to be addressed whether these actors are also required for the specification of the ventral habenula. However, as the ventral and a dorsal habenulae arise from different pool of progenitor at the thalamic-epithalamic border [16,69], it is clearly possible that their development requires a different combination of proneural genes and their upstream regulators.

## Supporting Information

S1 Fig Expression of *pax6a* and *Tg(neurog1*:*GFP)* co-localises in dorsal habenulae. Confocal sections (A-C) of the head of a *Tg(-8*.*4neurog1*:*GFP)*^*sb1*^ embryo at 36 hpf after whole-mount immunostaining against GFP (A, green), in situ hybridization against *pax6a* (B, red) and Immunostaining against HuC/D (D, magenta); cell nuclei staining (in blue) makes brain structures visible in A, B, D, and merges are shown in C (A+B) and E (A+D). The *Tg(-8*.*4neurog1*:*GFP)* transgene is expressed in the epithalamus, both in epiphyseal (ep) and habenular neurons (Hb, white brackets), as well as other brain structures such as the telencephalon (Tel) and the tectum (Tc). The expression of *Tg(-8*.*4neurog1*:*GFP)*^*sb1*^ recapitulates endogenous *neurog1* expression in habenular progenitors (described previously in [25]), although it can also be detected in newly-born HuC+ habenular neurons, probably due to persistence of the fluorescent reporter which acts as a short term lineage label (E). The expression of *pax6a* overlaps broadly with most of the Neurog1:GFP+ neurons in both the left and right habenulae (n = 15/15; C).
(TIF)

S2 Fig Sonic Hedgehog ligand (*Shha*) is required for habenular development. Whole-mount *in situ* hybridization against *cxcr4b* at 36 hpf (A,A’,B,B’) or *brn3a* at 48 hpf (C,C’,D,D’) showing heads (A-D) or the epithalamus (A’-D’) of wild type (A,A’,C,C’) or *shha*^*tbx392*^ embryos (B,B’,D,D’). While the expression of *cxcr4b* and *brn3a* is detected in the habenulae of all the wild type siblings (n = 8 and n = 14 respectively), the expression of both genes is strongly reduced in the epithalamus of *shha*^*tbx392*^ mutant embryos (B,B’, n = 12/13 and D,D’, n = 6/6). Embryos are viewed dorsally with anterior up.
(TIF)

S3 Fig Hh signalling is required for the expression of *pax6a*, *neurog1* and *neurod4* in the epithalamus. Whole-mount *in situ* hybridization against *pax6a* (A,B), *neurog1* (C,D) and *neurod4* (E,F) showing the head of control treated embryos (A, n = 10; C, n = 12; E, n = 8) or those treated from 16 hpf with cyclopamine (B, n = 10; D, n = 13; F, n = 9) in a lateral view of embryonic heads; all embryos are at 36 hpf and shown with anterior to the left. The expression of *pax6a*, *neurog1* and *neurod4* appears perturbed in the most dorsal part of the diencephalon subdivision (black brackets), while their expression does not appear significantly changed more ventrally or in other brain regions, such as the telencephalon (show as a *).
(TIF)

S4 Fig Hedgehog signalling is not required for *cxcr4b* expression in epiphysial projection neurons. Whole-mount *in situ* hybridization against *cxcr4b* at 28 hpf showing heads (A,B; lateral view) or the epithalamus (A’,B’; dorsal view with anterior up) of wild type (A,A’) or *smo*^*b641*^ embryos (B,B’). At 28 hpf, *cxcr4b* is expressed on both side of the epithalamic midline (*) in epiphysial projection neurons in both wild type siblings (n = 9/9) and *smo*^*b641*^ mutant embryos (n = 8/8). At this stage, *cxcr4b* is either only expressed in few left habenular neurons or not expressed yet in the habenulae. This expression could be detected in some wild type siblings (A’, black brackets, n = 3/9) but never in the habenulae of *smo*^*b641*^ mutant embryos (B’, n = 8/8).
(TIF)
