# Supplementary figures and images for: Habenular Neurogenesis in Zebrafish Is Regulated by a Hedgehog, Pax6 Proneural Gene Cascade
Source: PLoS One. 2016 Jul 7;11(7):e0158210. doi: 10.1371/journal.pone.0158210 (PMC4936704; doi:10.1371/journal.pone.0158210)

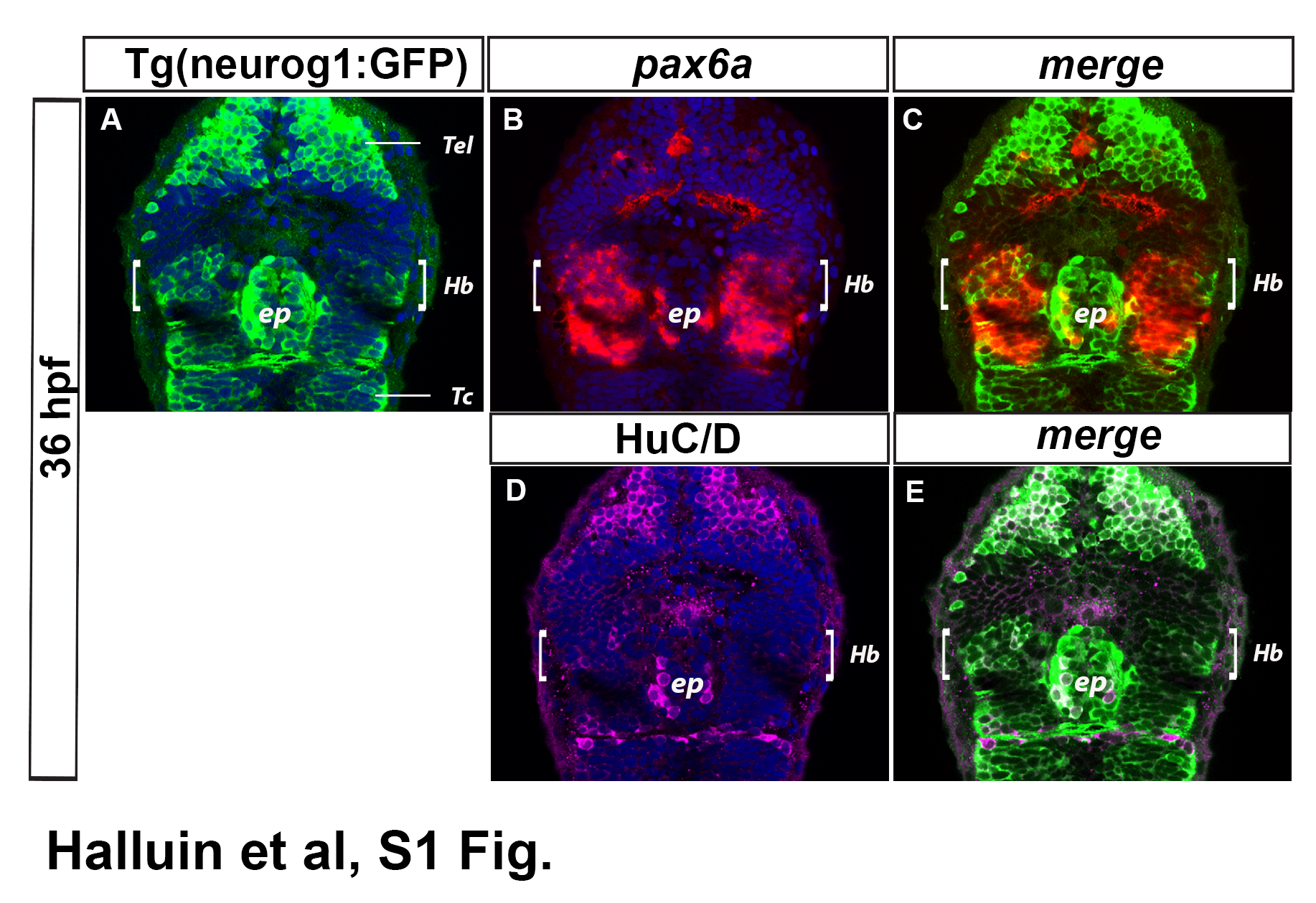

Supplement: S1 Fig — Confocal sections (A-C) of the head of a Tg(-8.4neurog1:GFP)sb1 embryo at 36 hpf after whole-mount immunostaining against GFP (A, green), in situ hybridization against pax6a (B, red) and Immunostaining against HuC/D (D, magenta); cell nuclei staining (in blue) makes brain structures visible in A, B, D, and merges are shown in C (A+B) and E (A+D). The Tg(-8.4neurog1:GFP) transgene is expressed in the epithalamus, both in epiphyseal (ep) and habenular neurons (Hb, white brackets), as well as other brain structures such as the telencephalon (Tel) and the tectum (Tc). The expression of Tg(-8.4neurog1:GFP)sb1 recapitulates endogenous neurog1 expression in habenular progenitors (described previously in [25]), although it can also be detected in newly-born HuC+ habenular neurons, probably due to persistence of the fluorescent reporter which acts as a short term lineage label (E). The expression of pax6a overlaps broadly with most of the Neurog1:GFP+ neurons in both the left and right habenulae (n = 15/15; C). (TIF) [file pone.0158210.s001.tif]

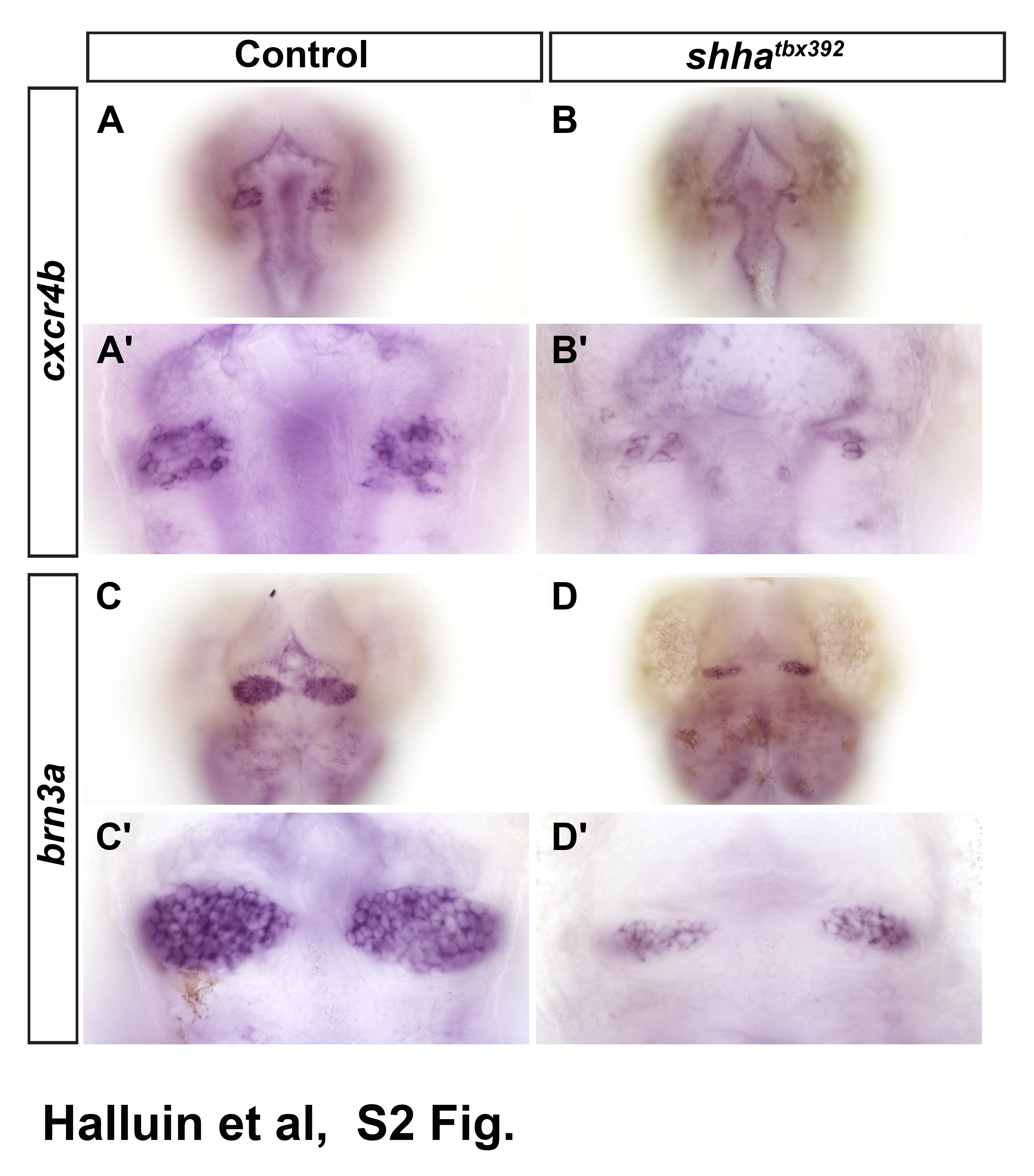

Supplement: S2 Fig — Whole-mount in situ hybridization against cxcr4b at 36 hpf (A,A’,B,B’) or brn3a at 48 hpf (C,C’,D,D’) showing heads (A-D) or the epithalamus (A’-D’) of wild type (A,A’,C,C’) or shhatbx392 embryos (B,B’,D,D’). While the expression of cxcr4b and brn3a is detected in the habenulae of all the wild type siblings (n = 8 and n = 14 respectively), the expression of both genes is strongly reduced in the epithalamus of shhatbx392 mutant embryos (B,B’, n = 12/13 and D,D’, n = 6/6). Embryos are viewed dorsally with anterior up. (TIF) [file pone.0158210.s002.tif]

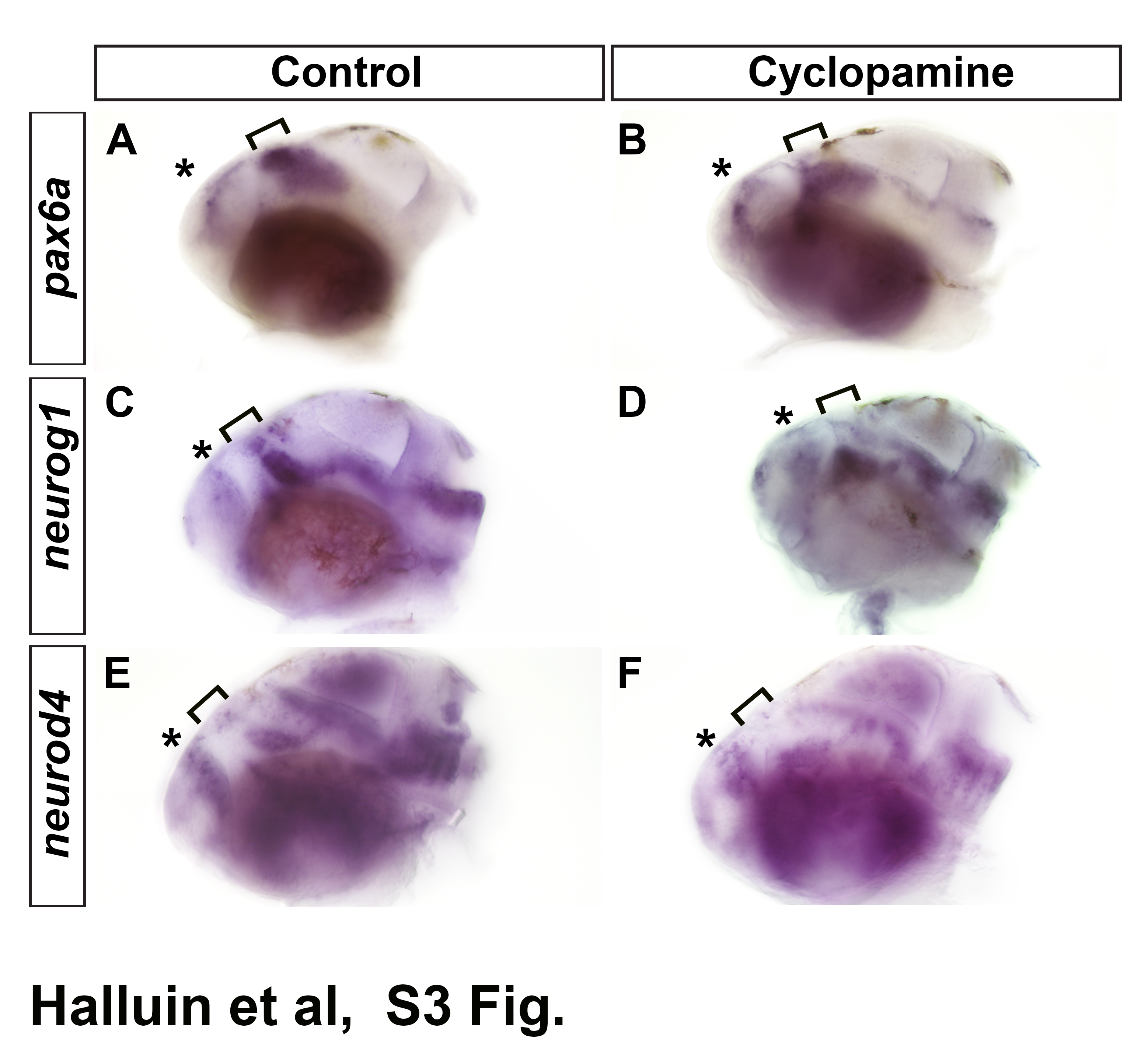

Supplement: S3 Fig — Whole-mount in situ hybridization against pax6a (A,B), neurog1 (C,D) and neurod4 (E,F) showing the head of control treated embryos (A, n = 10; C, n = 12; E, n = 8) or those treated from 16 hpf with cyclopamine (B, n = 10; D, n = 13; F, n = 9) in a lateral view of embryonic heads; all embryos are at 36 hpf and shown with anterior to the left. The expression of pax6a, neurog1 and neurod4 appears perturbed in the most dorsal part of the diencephalon subdivision (black brackets), while their expression does not appear significantly changed more ventrally or in other brain regions, such as the telencephalon (show as a *). (TIF) [file pone.0158210.s003.tif]

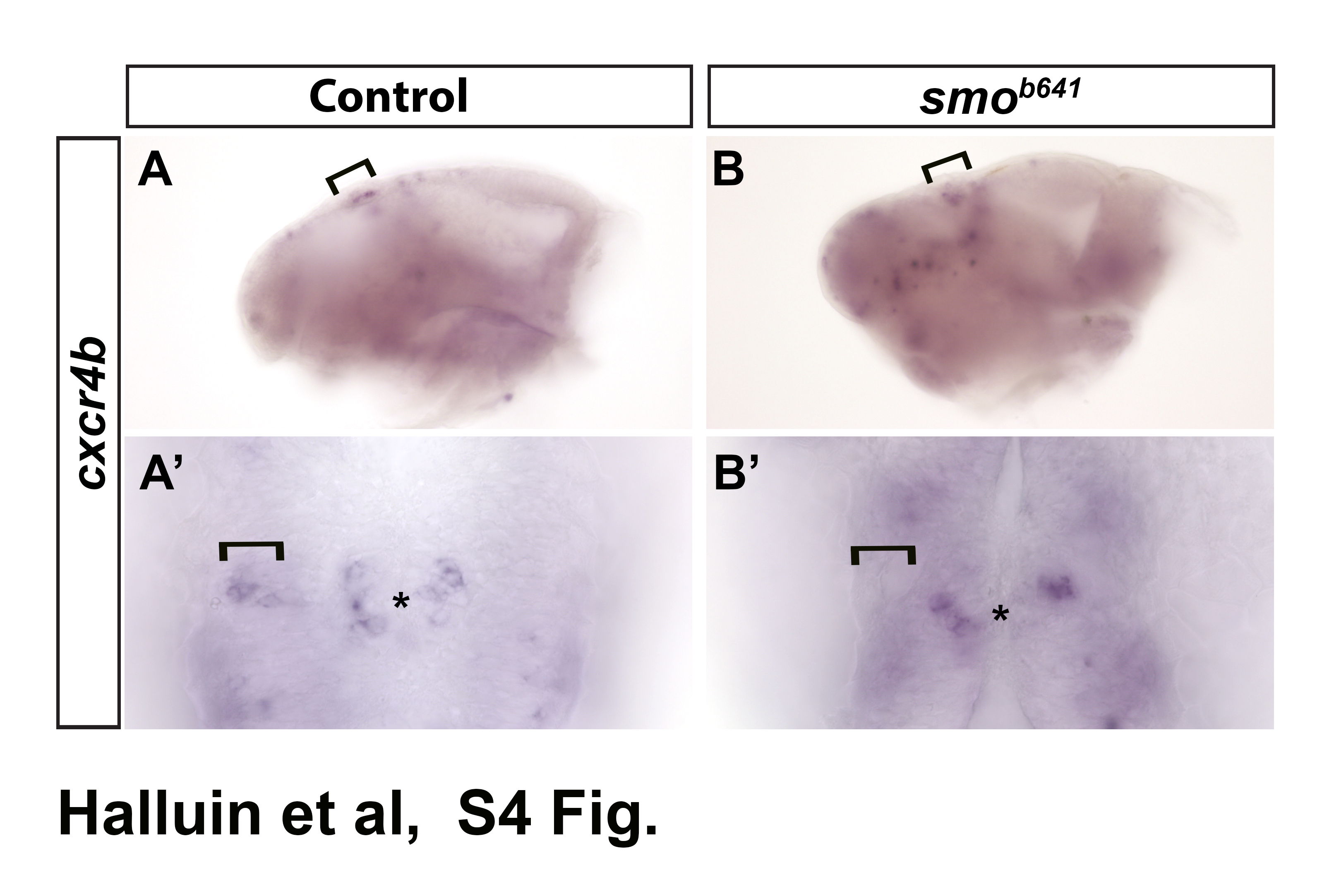

Supplement: S4 Fig — Whole-mount in situ hybridization against cxcr4b at 28 hpf showing heads (A,B; lateral view) or the epithalamus (A’,B’; dorsal view with anterior up) of wild type (A,A’) or smob641 embryos (B,B’). At 28 hpf, cxcr4b is expressed on both side of the epithalamic midline (*) in epiphysial projection neurons in both wild type siblings (n = 9/9) and smob641 mutant embryos (n = 8/8). At this stage, cxcr4b is either only expressed in few left habenular neurons or not expressed yet in the habenulae. This expression could be detected in some wild type siblings (A’, black brackets, n = 3/9) but never in the habenulae of smob641 mutant embryos (B’, n = 8/8). (TIF) [file pone.0158210.s004.tif]
